# Supplementary material for: Hot spot prediction in protein-protein interactions by an ensemble system
Source: BMC Syst Biol. 2018 Dec 31;12(Suppl 9):132. doi: 10.1186/s12918-018-0665-8 (PMC6311905; doi:10.1186/s12918-018-0665-8)
Supplement: Supplementary file 2 — All relevant MATLAB code. A simple Matlab implement of our predictor. (ZIP 19,742 kb) [file 12918_2018_665_MOESM2_ESM.zip › Additional file 2/Read ASA/ASA website/dbMPIKT/4KRMA.html]

 NetSurfP - Protein Surface Accessibility and Secondary Structure Predictions

  

|  |  |  |
| --- | --- | --- |
|  |  | NetSurfP - Protein Surface Accessibility and Secondary Structure Predictions  Technical University of Denmark |

  


---

```
# For publication of results, please cite:
# A generic method for assignment of reliability scores applied to solvent accessibility predictions.
# Bent Petersen, Thomas Nordahl Petersen, Pernille Andersen, Morten Nielsen and Claus Lundegaard
# BMC Structural Biology 2009, 9:51 doi:10.1186/1472-6807-9-51
#
# Column 1: Class assignment - B for buried or E for Exposed - Threshold: 25% exposure, but not based on RSA
# Column 2: Amino acid
# Column 3: Sequence name
# Column 4: Amino acid number
# Column 5: Relative Surface Accessibility - RSA
# Column 6: Absolute Surface Accessibility
# Column 7: Z-fit score for RSA prediction
# Column 8: Probability for Alpha-Helix
# Column 9: Probability for Beta-strand
# Column 10: Probability for Coil
E L  4KRMA                  1    0.675 123.574  -1.240   0.003   0.003   0.994
E E  4KRMA                  2    0.731 127.793   0.934   0.018   0.047   0.935
E E  4KRMA                  3    0.383  66.875  -0.114   0.005   0.015   0.979
E K  4KRMA                  4    0.624 128.295   0.050   0.005   0.015   0.979
E K  4KRMA                  5    0.600 123.502   0.702   0.018   0.088   0.893
E V  4KRMA                  6    0.317  48.646   0.672   0.004   0.138   0.858
B C  4KRMA                  7    0.068   9.505  -1.068   0.004   0.085   0.910
E N  4KRMA                  8    0.661  96.712  -0.480   0.004   0.085   0.910
E G  4KRMA                  9    0.464  36.533  -1.947   0.005   0.045   0.951
E I  4KRMA                 10    0.434  80.271  -0.252   0.018   0.019   0.964
E G  4KRMA                 11    0.403  31.716  -0.999   0.018   0.019   0.964
B I  4KRMA                 12    0.225  41.551  -1.409   0.018   0.047   0.935
E G  4KRMA                 13    0.416  32.771  -1.433   0.018   0.047   0.935
E E  4KRMA                 14    0.382  66.683  -2.284   0.018   0.047   0.935
B F  4KRMA                 15    0.233  46.783  -0.838   0.018   0.088   0.893
E K  4KRMA                 16    0.496 102.027  -1.649   0.018   0.047   0.935
E D  4KRMA                 17    0.449  64.715  -2.118   0.018   0.047   0.935
E S  4KRMA                 18    0.486  56.983  -1.933   0.053   0.043   0.903
E L  4KRMA                 19    0.446  81.589  -1.078   0.056   0.142   0.802
E S  4KRMA                 20    0.502  58.893  -0.725   0.064   0.216   0.721
B I  4KRMA                 21    0.204  37.685   0.145   0.056   0.142   0.802
E N  4KRMA                 22    0.369  53.992   0.319   0.018   0.088   0.893
B A  4KRMA                 23    0.248  27.330   0.105   0.430   0.016   0.555
E T  4KRMA                 24    0.647  89.683  -0.269   0.522   0.016   0.462
E N  4KRMA                 25    0.321  46.936   0.776   0.522   0.016   0.462
B I  4KRMA                 26    0.091  16.853   0.133   0.522   0.016   0.462
E K  4KRMA                 27    0.584 120.067   1.094   0.430   0.016   0.555
E H  4KRMA                 28    0.453  82.473   1.734   0.339   0.016   0.645
B F  4KRMA                 29    0.083  16.618  -0.292   0.184   0.043   0.773
E K  4KRMA                 30    0.512 105.236   1.217   0.052   0.084   0.864
E N  4KRMA                 31    0.591  86.552   0.493   0.018   0.047   0.935
B C  4KRMA                 32    0.064   9.000   1.149   0.005   0.262   0.733
B T  4KRMA                 33    0.265  36.825   0.359   0.002   0.816   0.182
E S  4KRMA                 34    0.268  31.421   1.079   0.001   0.959   0.040
B I  4KRMA                 35    0.120  22.126   0.795   0.001   0.959   0.040
E S  4KRMA                 36    0.391  45.872  -0.577   0.001   0.900   0.099
B G  4KRMA                 37    0.135  10.640  -1.283   0.003   0.718   0.279
B D  4KRMA                 38    0.250  36.025  -0.157   0.001   0.900   0.099
B L  4KRMA                 39    0.063  11.517   0.448   0.001   0.959   0.040
B H  4KRMA                 40    0.250  45.384   0.827   0.001   0.959   0.040
B I  4KRMA                 41    0.046   8.436   0.420   0.001   0.959   0.040
B L  4KRMA                 42    0.125  22.888   0.682   0.001   0.900   0.099
B P  4KRMA                 43    0.206  29.203   0.287   0.001   0.900   0.099
B V  4KRMA                 44    0.185  28.373  -0.151   0.021   0.756   0.223
B A  4KRMA                 45    0.259  28.531  -1.282   0.022   0.552   0.426
B F  4KRMA                 46    0.214  42.870  -0.789   0.021   0.451   0.528
E R  4KRMA                 47    0.491 112.531  -0.695   0.020   0.205   0.775
E G  4KRMA                 48    0.480  37.737  -2.231   0.018   0.047   0.935
E D  4KRMA                 49    0.514  74.082  -1.812   0.018   0.047   0.935
E S  4KRMA                 50    0.468  54.838  -1.693   0.052   0.084   0.864
E F  4KRMA                 51    0.431  86.562  -1.626   0.052   0.084   0.864
E T  4KRMA                 52    0.377  52.290  -0.944   0.056   0.142   0.802
E H  4KRMA                 53    0.568 103.283  -1.699   0.019   0.141   0.840
B T  4KRMA                 54    0.248  34.398  -1.250   0.018   0.047   0.935
E P  4KRMA                 55    0.483  68.538  -0.788   0.018   0.047   0.935
E P  4KRMA                 56    0.401  56.845  -0.948   0.052   0.084   0.864
E L  4KRMA                 57    0.350  64.158  -1.204   0.052   0.084   0.864
E D  4KRMA                 58    0.361  51.962   0.164   0.053   0.043   0.903
B P  4KRMA                 59    0.198  28.167  -1.585   0.184   0.043   0.773
E Q  4KRMA                 60    0.583 104.088  -0.661   0.184   0.043   0.773
E E  4KRMA                 61    0.534  93.290   0.193   0.268   0.043   0.689
B L  4KRMA                 62    0.106  19.354  -0.609   0.184   0.043   0.773
E D  4KRMA                 63    0.512  73.750  -0.599   0.184   0.043   0.773
E I  4KRMA                 64    0.390  72.243   0.833   0.184   0.043   0.773
B L  4KRMA                 65    0.080  14.630   0.305   0.058   0.017   0.925
E K  4KRMA                 66    0.640 131.586   0.596   0.058   0.017   0.925
E T  4KRMA                 67    0.473  65.577   1.432   0.053   0.043   0.903
B V  4KRMA                 68    0.061   9.422   0.710   0.064   0.216   0.721
E K  4KRMA                 69    0.359  73.908   0.851   0.022   0.552   0.426
E E  4KRMA                 70    0.354  61.861   0.512   0.018   0.846   0.136
B I  4KRMA                 71    0.063  11.655   0.542   0.018   0.846   0.136
B T  4KRMA                 72    0.215  29.848   0.472   0.021   0.756   0.223
B G  4KRMA                 73    0.138  10.861  -1.526   0.021   0.756   0.223
B F  4KRMA                 74    0.126  25.248   0.775   0.011   0.918   0.071
B L  4KRMA                 75    0.043   7.873   0.787   0.006   0.962   0.032
B L  4KRMA                 76    0.093  17.047   1.238   0.006   0.962   0.032
B I  4KRMA                 77    0.025   4.569   0.896   0.006   0.962   0.032
B Q  4KRMA                 78    0.118  21.111   0.419   0.011   0.918   0.071
B A  4KRMA                 79    0.289  31.826  -0.459   0.018   0.846   0.136
B W  4KRMA                 80    0.074  17.821  -0.468   0.021   0.451   0.528
E P  4KRMA                 81    0.478  67.857   0.296   0.019   0.141   0.840
E E  4KRMA                 82    0.612 106.864  -1.163   0.018   0.047   0.935
E N  4KRMA                 83    0.405  59.307  -1.219   0.053   0.043   0.903
E R  4KRMA                 84    0.281  64.280  -0.311   0.113   0.043   0.844
E T  4KRMA                 85    0.451  62.554  -0.613   0.184   0.043   0.773
B D  4KRMA                 86    0.229  33.028  -1.567   0.181   0.016   0.803
B L  4KRMA                 87    0.276  50.481  -0.873   0.181   0.016   0.803
E H  4KRMA                 88    0.528  96.043   0.328   0.257   0.016   0.727
B A  4KRMA                 89    0.206  22.745  -0.238   0.257   0.016   0.727
B F  4KRMA                 90    0.070  14.129   0.146   0.115   0.016   0.868
E E  4KRMA                 91    0.588 102.724   0.860   0.058   0.017   0.925
E N  4KRMA                 92    0.469  68.691   0.175   0.053   0.043   0.903
B L  4KRMA                 93    0.096  17.559   0.732   0.064   0.216   0.721
E E  4KRMA                 94    0.435  76.047   1.022   0.023   0.655   0.322
E I  4KRMA                 95    0.370  68.468   1.283   0.018   0.846   0.136
B I  4KRMA                 96    0.102  18.851   1.231   0.018   0.846   0.136
E R  4KRMA                 97    0.474 108.500  -0.197   0.022   0.552   0.426
B G  4KRMA                 98    0.227  17.896  -1.808   0.022   0.359   0.619
E R  4KRMA                 99    0.466 106.622   0.196   0.022   0.552   0.426
E T  4KRMA                100    0.422  58.490  -0.192   0.023   0.655   0.322
E K  4KRMA                101    0.274  56.362  -0.420   0.022   0.552   0.426
E Q  4KRMA                102    0.312  55.812  -0.373   0.069   0.386   0.545
E H  4KRMA                103    0.507  92.296  -1.351   0.056   0.142   0.802
E G  4KRMA                104    0.452  35.596  -1.588   0.118   0.150   0.732
B Q  4KRMA                105    0.138  24.700   0.027   0.079   0.592   0.329
B F  4KRMA                106    0.075  15.153  -1.119   0.085   0.775   0.140
B S  4KRMA                107    0.030   3.457  -0.128   0.072   0.855   0.073
B L  4KRMA                108    0.031   5.749   0.272   0.151   0.816   0.033
B A  4KRMA                109    0.031   3.394   0.016   0.151   0.816   0.033
B V  4KRMA                110    0.058   8.899   0.466   0.072   0.855   0.073
B V  4KRMA                111    0.226  34.798  -0.066   0.085   0.775   0.140
E S  4KRMA                112    0.270  31.679  -0.010   0.135   0.317   0.548
E L  4KRMA                113    0.446  81.718   0.036   0.053   0.043   0.903
E N  4KRMA                114    0.444  65.031   0.543   0.018   0.019   0.964
B I  4KRMA                115    0.067  12.339  -0.049   0.113   0.087   0.800
E T  4KRMA                116    0.503  69.808   0.910   0.199   0.152   0.649
E S  4KRMA                117    0.467  54.732   0.537   0.231   0.330   0.439
B L  4KRMA                118    0.095  17.321   0.664   0.216   0.235   0.548
E G  4KRMA                119    0.398  31.354  -0.351   0.191   0.086   0.723
B L  4KRMA                120    0.092  16.772   0.470   0.184   0.043   0.773
E R  4KRMA                121    0.482 110.309   1.031   0.268   0.043   0.689
B S  4KRMA                122    0.251  29.441   0.246   0.455   0.046   0.498
B L  4KRMA                123    0.090  16.442   0.005   0.701   0.107   0.192
E K  4KRMA                124    0.386  79.297   0.610   0.649   0.163   0.188
E E  4KRMA                125    0.399  69.758   1.116   0.578   0.229   0.194
B I  4KRMA                126    0.071  13.098   0.544   0.578   0.229   0.194
E S  4KRMA                127    0.378  44.278  -0.154   0.428   0.171   0.402
E D  4KRMA                128    0.539  77.699   0.015   0.113   0.043   0.844
B G  4KRMA                129    0.226  17.802  -1.891   0.053   0.043   0.903
B D  4KRMA                130    0.194  27.883  -0.505   0.066   0.296   0.638
B V  4KRMA                131    0.053   8.192  -0.594   0.021   0.756   0.223
B I  4KRMA                132    0.168  31.024   1.067   0.011   0.918   0.071
B I  4KRMA                133    0.065  11.932  -0.385   0.011   0.918   0.071
E S  4KRMA                134    0.354  41.430   0.627   0.021   0.756   0.223
E G  4KRMA                135    0.572  45.056  -0.110   0.022   0.359   0.619
B N  4KRMA                136    0.146  21.330   0.345   0.004   0.085   0.910
E K  4KRMA                137    0.427  87.875  -0.301   0.005   0.045   0.951
E N  4KRMA                138    0.441  64.577  -0.464   0.019   0.141   0.840
B L  4KRMA                139    0.048   8.880  -0.034   0.021   0.279   0.699
B C  4KRMA                140    0.057   7.989   0.207   0.021   0.451   0.528
B Y  4KRMA                141    0.209  44.663  -0.043   0.021   0.451   0.528
B A  4KRMA                142    0.226  24.927  -0.117   0.021   0.279   0.699
E N  4KRMA                143    0.488  71.414  -0.381   0.020   0.205   0.775
E T  4KRMA                144    0.460  63.788  -0.195   0.019   0.141   0.840
B I  4KRMA                145    0.235  43.568  -0.604   0.056   0.142   0.802
E N  4KRMA                146    0.499  72.995   0.002   0.113   0.087   0.800
B W  4KRMA                147    0.122  29.365  -0.136   0.191   0.086   0.723
E K  4KRMA                148    0.548 112.641   1.018   0.199   0.152   0.649
E K  4KRMA                149    0.480  98.736   0.692   0.199   0.152   0.649
B L  4KRMA                150    0.103  18.786  -0.082   0.199   0.152   0.649
B F  4KRMA                151    0.237  47.526  -0.419   0.118   0.150   0.732
E G  4KRMA                152    0.519  40.814   0.042   0.056   0.142   0.802
E T  4KRMA                153    0.455  63.136   0.051   0.018   0.047   0.935
E S  4KRMA                154    0.693  81.196  -1.187   0.018   0.019   0.964
E G  4KRMA                155    0.666  52.422  -1.284   0.018   0.047   0.935
E Q  4KRMA                156    0.321  57.331  -0.092   0.018   0.047   0.935
E K  4KRMA                157    0.535 110.070   0.104   0.005   0.262   0.733
B T  4KRMA                158    0.177  24.605  -0.071   0.018   0.846   0.136
B K  4KRMA                159    0.246  50.520   0.489   0.001   0.900   0.099
B I  4KRMA                160    0.092  17.057  -0.011   0.011   0.918   0.071
E I  4KRMA                161    0.287  53.076  -0.038   0.018   0.846   0.136
E S  4KRMA                162    0.454  53.232  -1.056   0.023   0.655   0.322
B N  4KRMA                163    0.295  43.188  -1.406   0.020   0.205   0.775
E R  4KRMA                164    0.490 112.187  -0.114   0.018   0.047   0.935
E G  4KRMA                165    0.630  49.565   0.341   0.018   0.019   0.964
E E  4KRMA                166    0.587 102.619  -0.413   0.058   0.017   0.925
E N  4KRMA                167    0.730 106.799   0.194   0.053   0.043   0.903
E S  4KRMA                168    0.499  58.530   0.935   0.113   0.043   0.844
B C  4KRMA                169    0.068   9.589  -2.132   0.113   0.043   0.844
E K  4KRMA                170    0.568 116.817   0.685   0.184   0.043   0.773
E A  4KRMA                171    0.765  84.281   0.551   0.113   0.043   0.844
E T  4KRMA                172    0.548  75.952  -0.032   0.058   0.017   0.925
E G  4KRMA                173    0.650  51.171  -1.348   0.005   0.015   0.979
E Q  4KRMA                174    0.583 104.142   0.166   0.005   0.015   0.979
B V  4KRMA                175    0.251  38.563  -0.206   0.005   0.045   0.951
B C  4KRMA                176    0.083  11.597  -0.951   0.005   0.045   0.951
E H  4KRMA                177    0.315  57.353   0.517   0.005   0.015   0.979
E A  4KRMA                178    0.689  75.873  -0.427   0.018   0.019   0.964
E L  4KRMA                179    0.377  69.029  -0.311   0.018   0.019   0.964
B C  4KRMA                180    0.045   6.332  -1.714   0.018   0.019   0.964
E S  4KRMA                181    0.356  41.700  -0.462   0.005   0.015   0.979
E P  4KRMA                182    0.689  97.812  -0.720   0.005   0.015   0.979
E E  4KRMA                183    0.528  92.311  -1.552   0.005   0.015   0.979
E G  4KRMA                184    0.310  24.428  -0.885   0.005   0.015   0.979
B C  4KRMA                185    0.056   7.876  -2.917   0.018   0.047   0.935
E W  4KRMA                186    0.335  80.568  -0.077   0.005   0.045   0.951
E G  4KRMA                187    0.420  33.030  -0.993   0.005   0.015   0.979
E P  4KRMA                188    0.624  88.574  -1.148   0.003   0.003   0.994
E E  4KRMA                189    0.506  88.381  -0.333   0.003   0.003   0.994
E P  4KRMA                190    0.421  59.797  -0.364   0.053   0.005   0.942
E R  4KRMA                191    0.715 163.689   0.353   0.176   0.004   0.820
E D  4KRMA                192    0.339  48.879   0.957   0.181   0.016   0.803
B C  4KRMA                193    0.063   8.887  -1.192   0.216   0.235   0.548
E V  4KRMA                194    0.331  50.844   0.951   0.148   0.418   0.435
E S  4KRMA                195    0.430  50.443   0.843   0.252   0.423   0.325
B C  4KRMA                196    0.052   7.329   0.422   0.135   0.317   0.548
E R  4KRMA                197    0.415  95.058   1.442   0.125   0.227   0.648
E N  4KRMA                198    0.476  69.760  -0.192   0.064   0.216   0.721
B V  4KRMA                199    0.121  18.582   0.995   0.069   0.386   0.545
E S  4KRMA                200    0.445  52.107   0.241   0.021   0.451   0.528
E R  4KRMA                201    0.349  79.829   0.331   0.021   0.279   0.699
E G  4KRMA                202    0.668  52.611  -0.219   0.005   0.015   0.979
E R  4KRMA                203    0.632 144.820   0.004   0.005   0.015   0.979
E E  4KRMA                204    0.414  72.273   1.667   0.021   0.451   0.528
B C  4KRMA                205    0.051   7.146  -0.108   0.023   0.655   0.322
B V  4KRMA                206    0.200  30.694   1.179   0.022   0.552   0.426
E D  4KRMA                207    0.615  88.607  -0.394   0.021   0.451   0.528
E K  4KRMA                208    0.555 114.225   1.428   0.020   0.205   0.775
B H  4KRMA                209    0.073  13.279   0.231   0.005   0.015   0.979
E H  4KRMA                210    0.461  83.892   0.423   0.005   0.015   0.979
E H  4KRMA                211    0.725 131.968   0.217   0.005   0.015   0.979
E H  4KRMA                212    0.567 103.192  -1.337   0.005   0.045   0.951
E H  4KRMA                213    0.533  96.935  -0.423   0.018   0.047   0.935
E H  4KRMA                214    0.639 116.143  -1.381   0.003   0.003   0.994

Explain the output.  Go back.
```

---
